# Rhodoptilometrin, a Crinoid-Derived Anthraquinone, Induces Cell Regeneration by Promoting Wound Healing and Oxidative Phosphorylation in Human Gingival Fibroblast Cells

**DOI:** 10.3390/md17030138

**Published:** 2019-02-27

**Authors:** Chung-Chih Tseng, Yu-Cheng Lai, Tsu-Jen Kuo, Jui-Hsin Su, Ping-Jyun Sung, Chien-Wei Feng, Yen-You Lin, Pei-Chin Chen, Ming-Hong Tai, Shu-Yu Cheng, Hsiao-Mei Kuo, Zhi-Hong Wen

**Affiliations:** 1 Department of Marine Biotechnology and Resources, National Sun Yat-sen University, Kaohsiung 80424, Taiwan; caviton@gmail.com (C.-C.T.); yclai@vghks.gov.tw (Y.-C.L.); tjkuo@vghks.gov.tw (T.-J.K.); qscjuejuejue@gmail.com (C.-W.F.); peichin1128@gmail.com (P.-C.C.); joygetit@gmail.com (S.-Y.C.); 2 Department of Dentistry, Zuoying Branch of Kaohsiung Armed Forces General Hospital, Kaohsiung 81357, Taiwan; 3 Department of Orthopedics, Kaohsiung Veterans General Hospital, Kaohsiung 81362, Taiwan; 4 Department of Stomatology, Kaohsiung Veterans General Hospital, Kaohsiung 81362, Taiwan; 5 Graduate Institute of Marine Biology, National Dong Hwa University, Pingtung 944, Taiwan; x2219@nmmba.gov.tw (J.-H.S.); pjsung@nmmba.gov.tw (P.-J.S.); 6 National Museum of Marine Biology and Aquarium, Pingtung County 94450, Taiwan; 7 Department of Orthopedic Surgery, Ping-Tung Christian Hospital, Pingtung 90059, Taiwan; chas6119@gmail.com; 8 Doctoral Degree Program in Marine Biotechnology, National Sun Yat-sen University, Kaohsiung 80424, Taiwan; 9 Center for Neuroscience, National Sun Yat-sen University, Kaohsiung 80424, Taiwan; minghongtai@gmail.com; 10 Institute of Biomedical Sciences, National Sun Yat-sen University, Kaohsiung 80424, Taiwan

**Keywords:** gingival recession, oral mucosa fibroblasts, extracellular matrix, mitochondria, oxidative phosphorylation

## Abstract

Gingival recession (GR) potentially leads to the exposure of tooth root to the oral cavity microenvironment and increases susceptibility to dental caries, dentin hypersensitivity, and other dental diseases. Even though many etiological factors were reported, the specific mechanism of GR is yet to be elucidated. Given the species richness concerning marine biodiversity, it could be a treasure trove for drug discovery. In this study, we demonstrate the effects of a marine compound, (+)-rhodoptilometrin from crinoid, on gingival cell migration, wound healing, and oxidative phosphorylation (OXPHOS). Experimental results showed that (+)-rhodoptilometrin can significantly increase wound healing, migration, and proliferation of human gingival fibroblast cells, and it does not have effects on oral mucosa fibroblast cells. In addition, (+)-rhodoptilometrin increases the gene and protein expression levels of focal adhesion kinase (FAK), fibronectin, and type I collagen, changes the intracellular distribution of FAK and F-actin, and increases OXPHOS and the expression levels of complexes I~V in the mitochondria. Based on our results, we believe that (+)-rhodoptilometrin might increase FAK expression and promote mitochondrial function to affect cell migration and promote gingival regeneration. Therefore, (+)-rhodoptilometrin may be a promising therapeutic agent for GR.

## 1. Introduction

Gingival recession (GR) is a pathological state in which the loss of gum tissue or a retraction of the gingival margin results in the exposure of the roots of the teeth. GR leads to dental caries [1], dentin hypersensitivity [2], and aesthetic problems [3]. There are many potential risk factors for GR, including age [3], genetic factors [4,5], pathological factors [6,7], anatomical factors [8], and chronic trauma [9,10]. GR can be treated with crown restoration by prosthodontic prosthesis, composite resin filling, or using autologous oral soft tissue grafting for gingival reconstruction surgery [11]. In addition, desensitizing agents such as fluoride varnish, fluorine, or fluoride paste can help reduce the sensitivity of the tooth root [12]. To date, protective mechanisms of GR remain to be elucidated, and there is no specific therapeutic drug available. However, persistent salivation in the oral cavity and invasion by microorganisms may interfere or delay wound-healing speed, causing a challenge in the use of drugs for treating oral wounds. Therefore, elucidation of the mechanism for improving wound-healing capabilities is still an important topic. Furthermore, many previous studies on oral wounds found that promoting cell proliferation and migration, and enhancing wound-healing speed will aid in the development of drugs for oral diseases [13,14].

Cell migration refers to the movement of cells after receiving migration signals or sensing of certain chemicals. Cell migration involves multiple simultaneous dynamic processes, such as a cycle of surface adhesion and release, cell mobility and maintenance, development and destruction of pseudopodium (including lamellipodium and filopodia), and interactions with environmental cues such as the extracellular matrix (ECM), chemicals, or other cells in the environment, which will aid in this dynamic process [15,16,17]. Also, cell migration is dependent on extracellular signals, intracellular signal transduction, cytoskeleton, motor proteins, and focal adhesions [18,19]. Focal adhesion kinase (FAK) is an intracellular protein-tyrosine kinase that plays a significant role in cell signaling and is regarded as a central regulator of integrin signaling that regulates the assembly and disassembly of focal adhesion during cell migration [20]. After tissue damage, tissue remodeling during the wound-healing phase causes damaged tissues to be replaced. During this stage, fibroblasts play an important role as they proliferate, differentiate, and migrate to refill the damaged tissue. In the processes mentioned earlier, cell migration is a crucial biological reaction in wound healing [21,22,23], in which the ECM plays an important role [24]. During tissue repair, cells secrete ECM, such as collagen, fibronectin, and proteoglycans [25], which aid in filling the damaged tissue, enabling cells to migrate and reconstruct tissues. Moreover, ECM acts as a scaffold to support the entire cell, and can regulate gene expression, cell proliferation, and cell migration [25]. Therefore, corresponding cells and a healthy ECM are needed to establish wound healing and tissue regeneration conditions.

The mitochondrion is a double-membrane organelle, and its primary function is to convert carbohydrates to ATP to provide energy to cells. Mitochondria are the energy factories for respiration in cells. In aerobic respiration, pyruvate produces ATP via the tricarboxylic acid (TCA) cycle and oxidative phosphorylation in mitochondria, and is reduced to lactate in anaerobic respiration and produces ATP in the cytoplasm. In addition, the five complexes I~V in the electron transport chain in the inner mitochondrial membranes are associated with ATP generation. Evidence shows that mitochondria play an important role in cell mobility (adhesion, migration, proliferation, and differentiation), signal transduction, and cellular responses to external stimuli [26]. A study showed that changes in mitochondrial function can affect ECM remodeling; on the contrary, a deficiency in collagen will affect mitochondrial function [27]. Another study showed that FAK, which acts as a hub for cell migration, will affect lactate production during respiration [28]. Furthermore, ECM components, such as vitronectin, laminin, fibronectin, etc., were reported to stimulate mitochondrial function [29]. Thus, it becomes clear that mitochondrial function is associated not only with energy production, but also with cell migration.

According to previous studies, it was proven that marine natural products with great developmental potential can usually be found in algae, corals, sponges, ascidians, and other marine organisms [30,31,32]. Therefore, marine organisms hold great potential for the development of future drugs. The marine natural compound rhodoptilometrin is an anthraquinone that was extracted and purified from *Himerometra magnipinna* in 1967 [33]. In a previous study conducted in 2009, Wright et al. employed nuclear magnetic resonance (NMR) to prove that rhodoptilometrin exists as two stereoisomers (*S*- or *R*-configuration) [34]. In a previous study, we employed X-ray crystallography to confirm that the rhodoptilometrin isolated from *H. magnipinna* is in the *R*-configuration, i.e., (+)-rhodoptilometrin [35]. A previous study showed that (+)-rhodoptilometrin exhibits significant cytotoxicity toward the MCF-7 breast cancer cell line, SF-268 glioblastoma cell line, and the H460 non-small-cell lung cancer cell line [34]. In a previous study, we found that (+)-rhodoptilometrin showed significant concentration-dependent inhibitory effects on nitric oxide synthase in lipopolysaccharide-stimulated murine RAW 264.7 macrophages [35,36]. In this study, we analyzed the effects of (+)-rhodoptilometrin on gingival cell proliferation, wound healing, cell migration, and mitochondrial function in hopes that (+)-rhodoptilometrin can be used as a drug for treating GR.

## 2. Results

### 2.1. Effects of (+)-Rhodoptilometrin on Wound Healing, Cell Viability, and Cell Migration in hGF-1 Cells

**Figure 1:**
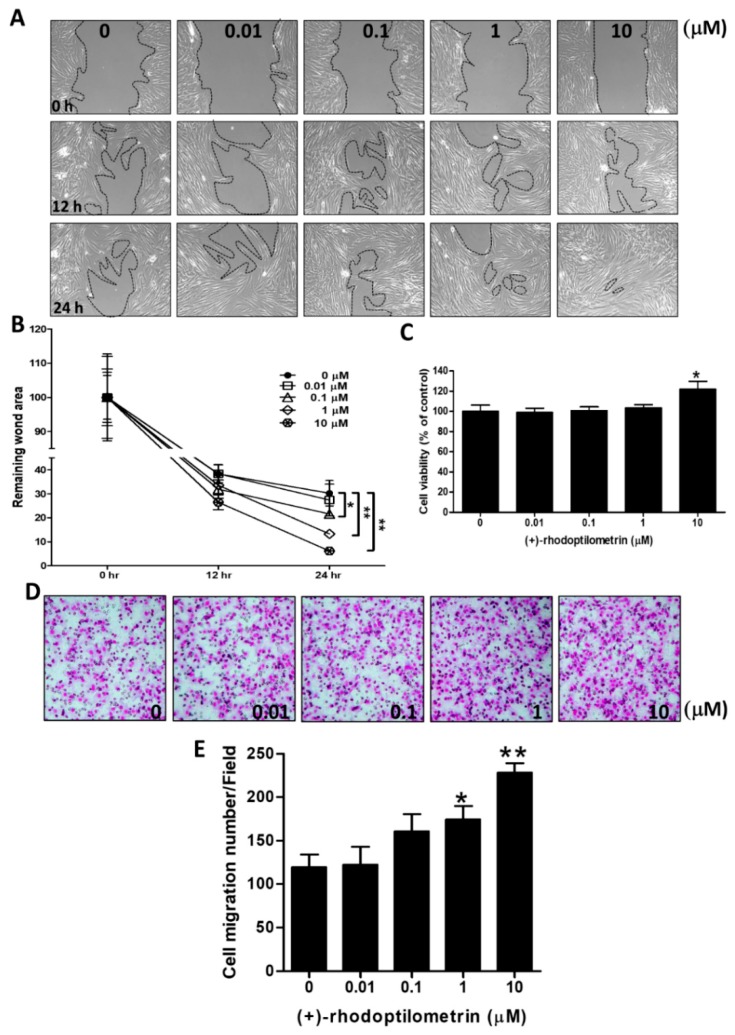
Effects of various concentrations of (+)-rhodoptilometrin treatment on the cell viability, cell migration, and wound healing of hGF-1 cells. (**A**) Cells were treated with scratch and the indicated concentrations of (+)-rhodoptilometrin for 0, 12, and 24 h, and then photographed by phase-contrast microscopy at 100× magnification. (**B**) Scratch-test assay statistics of the remaining wound area were normalized with the time point 0 h. The results are expressed as means ± standard error of the mean (SEM) of three independent experiments. (**C**) Cells were treated with an increasing concentration of (+)-rhodoptilometrin for 24 h, and then a 3-(4,5-dimethylthiazol-2-yl)-2,5-diphenyl tetrazolium bromide (MTT) assay was performed to measure cell viability. Cell viability (%) is expressed as a percentage compared to the untreated cells. The results are expressed as means ± SEM of three independent experiments. (**D**) The profile of migration cells treated with (+)-rhodoptilometrin of various doses for 24 h before being evaluated for chemotactic potency. The photographs present the cell migration morphologies. (**E**) Quantification of migration assay. The migrated cells were counted and calculated. Data (means ± SEM) are representative of at least three independent experiments. Student’s *t*-test determined the significance; * *p* < 0.05, ** *p* < 0.01, compared with untreated cells.

The scratch-test assay was used to analyze the effects of (+)-rhodoptilometrin on wound healing in hGF-1 cells. The experiment results showed that better wound healing could be observed in hGF-1 cells after culturing with (+)-rhodoptilometrin for 24 h compared with the control group cells (Figure 1A). After being used for quantitative analysis of the wound area, results showed that there were no significant changes in wound healing in hGF-1 cells after treatment with 1 and 10 μM for 12 h compared with the control group cells. The wound healing was significantly accelerated in hGF-1 cells that were treated with 0.01, 0.1, 1, and 10 μM (+)-rhodoptilometrin for 24 h compared with the control group cells (Figure 1B). The 3-(4,5-dimethylthiazol-2-yl)-2,5-diphenyl tetrazolium bromide (MTT) assay was used to analyze the viability of hGF-1 cells after treatment with different (+)-rhodoptilometrin concentrations. The experimental results showed that a concentration of 10 μM (+)-rhodoptilometrin significantly increased the viability of hGF-1 cells by 21.7% compared with the control group cells (Figure 1C). The transwell migration assay was used to analyze the effects of (+)-rhodoptilometrin on migration in hGF-1 cells. The experimental results showed that hGF-1 cells treated with (+)-rhodoptilometrin promoted a cell migration profile (Figure 1D). From quantitative analyses of the number of migrated cells, we found that there were no significant effects of treatment with 0.01 and 0.1 μM (+)-rhodoptilometrin on hGF-1 cell migration compared with the control group’s cell migration, however, treatment with 1 or 10 μM (+)-rhodoptilometrin significantly increased the number of migrated hGF-1 cells (Figure 1E). These results confirmed that (+)-rhodoptilometrin increases wound healing, cell viability, and cell migration in human gingival fibroblast cells.

### 2.2. Effects of (+)-Rhodoptilometrin on Wound Healing, Cell Viability, and Cell Migration in Oral Mucosa Fibroblast (OMF) Cells

**Figure 2:**
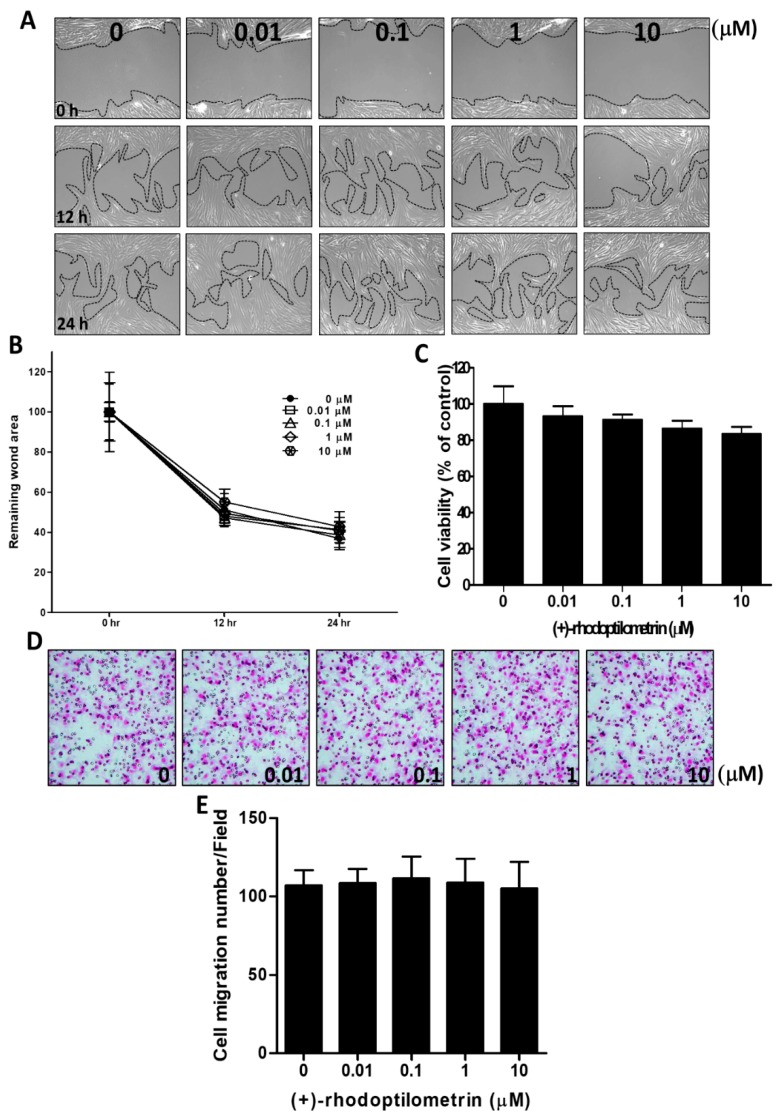
Effects of various concentrations of (+)-rhodoptilometrin treatment on the cell viability, cell migration, and wound healing of oral mucosa fibroblast (OMF) cells. (**A**) The cells were treated with an in vitro scratch assay and different concentrations of (+)-rhodoptilometrin for 0, 12, and 24 h, and then photographed by phase-contrast microscopy at 100× magnification. (**B**) Scratch-test assay statistics of the remaining wound area were normalized with the time point 0 h. The results are expressed as means ± SEM of three independent experiments. (**C**) Cells were treated with an increasing concentration of (+)-rhodoptilometrin for 24 h, and then an MTT assay was performed to measure cell viability. Cell viability (%) is expressed as a percentage compared to the untreated cells. The results are expressed as means ± SEM of three independent experiments. (**D**) The profile of migration cells treated with (+)-rhodoptilometrin of various doses for 24 h before being evaluated for chemotactic potency. The photographs present the cell migration morphologies. (**E**) Quantification of migration assay. The migrated cells were counted and calculated. Data (means ± SEM) are representative of at least three independent experiments.

The scratch-test assay was used to analyze the effects of (+)-rhodoptilometrin on wound healing in OMF cells. The experimental results showed that (+)-rhodoptilometrin had no significant effects on wound healing in OMF cells compared with the control group cells (Figure 2A). After quantitative analysis of the wound region, we found that there was no significant difference in wound healing on OMF cells treated with various concentrations of (+)-rhodoptilometrin and the control group cells (Figure 2B). The MTT assay was used to analyze the viability of OMF cells treated with different concentrations of (+)-rhodoptilometrin for 24 h. The experimental results showed that concentrations of 0, 0.01, 0.1, 1, and 10 μM (+)-rhodoptilometrin had no significant effects on the viability of OMF cells (Figure 2C). The transwell migration assay was used to analyze the effects of (+)-rhodoptilometrin on migration in OMF cells. Moreover, there was no significant difference in the migration of OMF cells treated with (+)-rhodoptilometrin when compared with the control group cells (Figure 2D). After quantitative analysis, we found that there were no significant differences in the number of migrated OMF cells treated with (+)-rhodoptilometrin and that of the control group cells (Figure 2E). These results suggested that (+)-rhodoptilometrin does not affect wound healing, cell viability, and cell migration in oral mucosa fibroblast cells.

### 2.3. Effects of (+)-Rhodoptilometrin on the Gene and Protein Expression Levels of FAK, Fibronectin, and Type I Collagen

**Figure 3:**
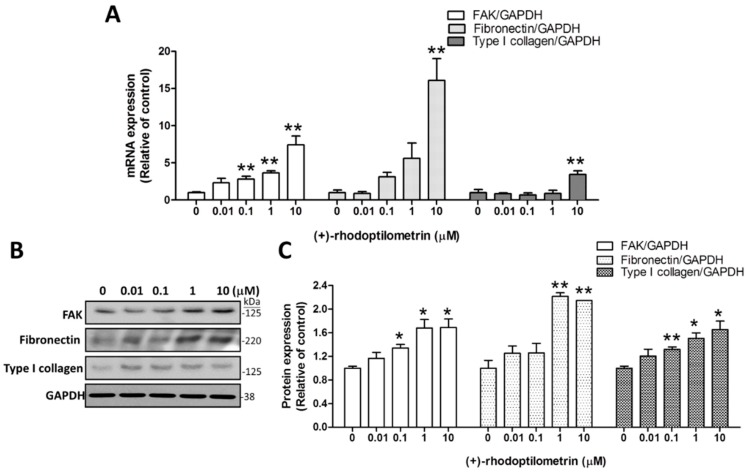
Effects of (+)-rhodoptilometrin on the messenger RNA (mRNA) and protein expression of focal adhesion kinase (FAK), fibronectin, and type I collagen in hGF-1 cells. (**A**) The expression of FAK, fibronectin, and type I collagen mRNA in hGF-1 cells was determined by qRT-PCR analysis after treatment with various dosages of (+)-rhodoptilometrin for 24 h. The relative FAK, fibronectin, and type I collagen mRNA levels were calculated from the ratio of FAK, fibronectin, and type I collagen over that of an internal control *GAPDH* gene. (**B**) FAK, fibronectin, and type I collagen expression in hGF-1 cells were evaluated by western blot analysis after treatment with various dosages of (+)-rhodoptilometrin for 24 h. Glyceraldehyde 3-phosphate dehydrogenase (GAPDH) was detected as an internal control. (**C**) The FAK, fibronectin, and type I collagen levels were quantified by protein band densitometry analysis using ImageJ software and normalized with the level of GAPDH. Data (means ± SEM) are representative of at least three independent experiments. Student’s *t*-test determined the significance; * *p* < 0.05, ** *p* < 0.01, compared with untreated cells.

Quantitative RT-PCR was used to quantify the effects of (+)-rhodoptilometrin on the expression levels of genes associated with migration (FAK, fibronectin, type I collagen) in hGF-1 cells. FAK is a focal adhesion-associated protein kinase and is a member of the focal adhesion protein family. FAK is responsible for cell–extracellular matrix connections and participates in cell adhesion and mobility. The experimental results showed that the FAK gene expression level of cells cultured with 0.1, 1, and 10 μM (+)-rhodoptilometrin were significantly higher than that in the control group cells. Likewise, fibronectin is a glycoprotein that is part of the extracellular matrix (ECM) and participates in cell migration, adhesion growth, and differentiation. The results showed that, in cells treated with 10 μM (+)-rhodoptilometrin, the gene expression level of fibronectin was significantly increased compared with the control group cells. In addition, type I collagen is a glycoprotein and is also part of the ECM and participates in cell migration. The experimental results showed that treatment with 10 μM (+)-rhodoptilometrin significantly increased the gene expression level of type I collagen compared with that in the control group cells; here, GAPDH was detected as an internal control (Figure 3A). Western blotting profile was used to quantify the effects of (+)-rhodoptilometrin on the protein expression levels of markers associated with migration including FAK (125 kDa), Fibronectin (220 kDa), and type I collagen (125 kDa) in hGF-1 cells, western bolt protein band profile with glyceraldehyde 3-phosphate dehydrogenase (GAPDH) as an internal control (Figure 3B). The quantitative results showed that treatment with 0.1, 1, and 10 μM of (+)-rhodoptilometrin significantly increased the protein expression levels of FAK and type I collagen compared with the control group. The treatment with 1 or 10 μM (+)-rhodoptilometrin significantly increased the protein expression level of fibronectin compared with the control group cells (Figure 3C). The above results showed that (+)-rhodoptilometrin can increase the gene and protein expression levels of markers associated with cell migration.

### 2.4. Effects of (+)-Rhodoptilometrin on the Distribution of FAK, and F-Actin Protein Expression

**Figure 4:**
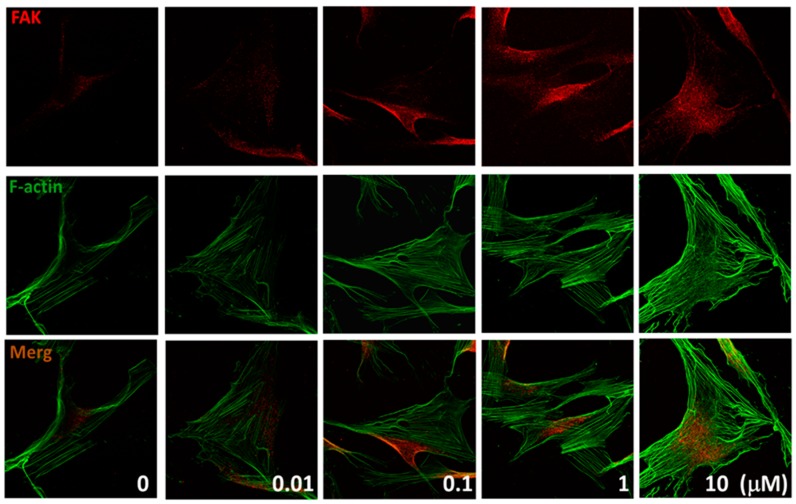
(+)-Rhodoptilometrin induced FAK and F-actin expression in hGF-1 cells. Immunofluorescence double staining was used to evaluate the distribution of FAK and F-actin protein. Cells were pretreated with an increasing concentration of (+)-rhodoptilometrin for 24 h and incubated with FAK antibodies one hour before harvest. They were then stained with Alexa 546-conjugated secondary antibodies (red) and Alexa Fluor 488 Phalloidin (green). The immunofluorescence profile was visualized under a confocal fluorescence microscope (1000×).

Immunofluorescence was used to evaluate the effects of different (+)-rhodoptilometrin concentrations (0.01, 0.1, 1, and 10 μM) on the distribution of FAK and F-actin (actin filaments) in hGF-1 cells, and this was observed using a confocal microscope. FAK and F-actin protein expression levels are associated with cell migration. The experiment results showed that, in the control group, FAK was present throughout the cell. However, after treatment with (+)-rhodoptilometrin, FAK (red color) was localized to the focal adhesions around the cells. Moreover, F-actin (green color) proteins were observed to be arranged in dense parallel arrays of bundles (Figure 4). The experimental results showed that (+)-rhodoptilometrin can increase the protein expression of FAK and F-actin and promote cell migration.

### 2.5. Effects of the FAK Inhibitor PF-562271 on Reverse Wound Healing in hGF-1 Cells Caused by (+)-Rhodoptilometrin

**Figure 5:**
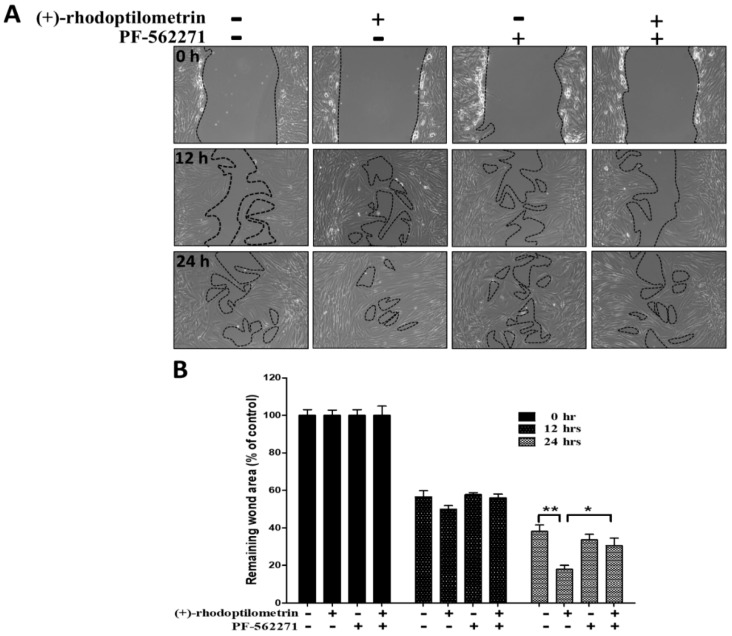
Effect of FAK inhibitor, PF-562271, on (+)-rhodoptilometrin-promoted wound healing in the hGF-1 cell. (**A**) After prior incubation with PF-562271 (10 μM) for 2 h, the cells were treated with scratch and (+)-rhodoptilometrin (10 μM) for 0, 12, and 24 h, and then photographed by phase-contrast microscopy at 100× magnification. (**B**) Scratch-test assay statistics of the remaining wound area were normalized with the time point 0 h. Data (means ± SEM) are representative of at least three independent experiments. Student’s *t*-test determined the significance; * *p* < 0.05, ** *p* < 0.01, compared with untreated cells in same time point.

The scratch-test assay was used to analyze the effects of the FAK inhibitor PF-562271 on increased wound healing in hGF-1 cells caused by (+)-rhodoptilometrin. The experiment results showed that the addition of PF-562271 decreases the wound-healing promoting effects of (+)-rhodoptilometrin on hGF-1 cells (Figure 5A). Results of the quantitative analysis of the wound region showed that there were no significant differences in the remaining wound area in control, (+)-rhodoptilometrin, PF-562271, and (+)-rhodoptilometrin + PF-562271 groups after 12 h of culture. However, after 24 h of culture, the remaining wound area of the (+)-rhodoptilometrin + PF-562271 group was significantly higher than that of the (+)-rhodoptilometrin group. From the experiment results, it can be observed that (+)-rhodoptilometrin can promote substantial wound healing, whereas PF-562271 administration significantly suppresses (+)-rhodoptilometrin-mediated promotion of wound-healing effects (Figure 5B).

### 2.6. (+)-Rhodoptilometrin Promotes Mitochondrial Oxidative Phosphorylation (OXPHOS) and Glycolytic Function in hGF-1 Cells

**Figure 6:**
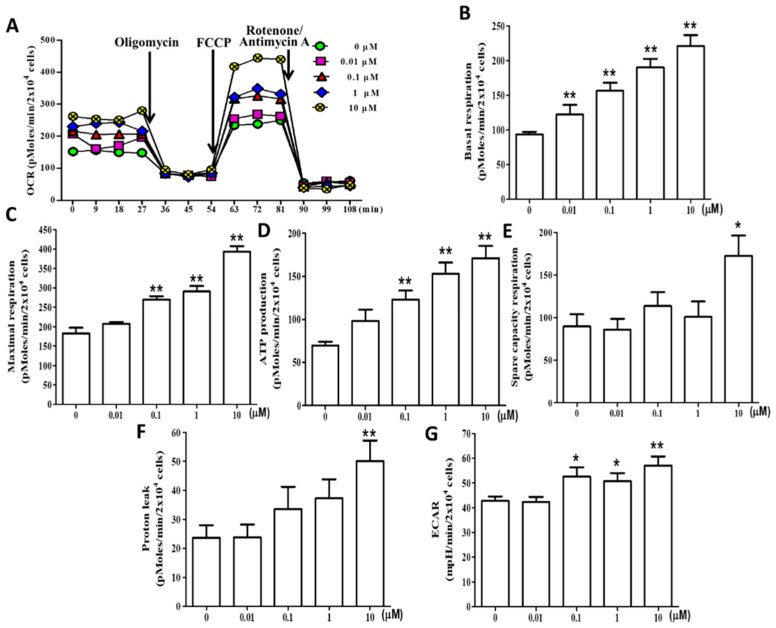
Effect of different concentrations of (+)-rhodoptilometrin on mitochondrial oxidative phosphorylation (OXPHOS) and glycolytic function in hGF-1 cells. (**A**) Time course (minutes) and OCR curve plot in hGF-1 cells with (+)-rhodoptilometrin treatment at different concentrations (0, 0.01, 0.1, 1, and 10 μM) for 24 h; cells were examined using the Seahorse Bioscience XF24 analyzer with or without oligomycin, carbonyl cyanide-4-(trifluoromethoxy) phenylhydrazone (FCCP), and rotenone/antimycin A to measure oxygen consumption rate (OCR; pMoles/min/2 × 10^4^ cells). Quantification of transformation in parameters induced by (+)-rhodoptilometrin treatment include the (**B**) basal respiration, (**C**) maximal respiration, (**D**) ATP production, (**E**) spare capacity respiration, and (**F**) proton leak. (**G**) Extracellular acidification rate (ECAR; mpH/min/2 × 10^4^ cells), which indicates the lactate production, was analyzed with Seahorse XF24. OCR and ECAR levels are quantified by normalization of cell numbers. Data (means ± SEM) are representative of at least three independent experiments. Student’s *t*-test determined the significance; * *p* < 0.05, ** *p* < 0.01, compared with untreated cells.

To assess the effects of (+)-rhodoptilometrin on OXPHOS and glycolytic function, we used a Seahorse Bioscience XF24 analyzer to measure the oxygen consumption rate (OCR) and extracellular acidification rate (ECAR) of hGF-1 cells. The OCR and measurement time (minutes) plots show that the addition of (+)-rhodoptilometrin increased OCR in hGF-1 cells. In hGF-1 cells treated with various concentrations of (+)-rhodoptilometrin overnight, the basal respiration OCR was measured four times under the conditions of cell function, and then 1 μM oligomycin, 0.5 μM carbonyl cyanide-4-(trifluoromethoxy) phenylhydrazone (FCCP), and 1 μM rotenone/antimycin A were continuously added to detect changes in respiration OCR at various stages (Figure 6A). Kuo et al. reported several mitochondrial respiratory stages of oxygen consumption rate (OCR) after the continuous addition of oligomycin, FCCP, and rotenone respiratory inhibitors to inhibit the electron transport chain [37]. The experiment results showed that treatment with 0.01, 0.1, 1, and 10 μM (+)-rhodoptilometrin significantly increased basal respiration in hGF-1 cells compared with the control group cells (Figure 6B); treatment with 0.1, 1, and 10 μM (+)-rhodoptilometrin significantly increased maximum respiration (Figure 6C) and ATP production (Figure 6D) in hGF-1 cells compared with the control group cells. Furthermore, (+)-rhodoptilometrin (10 μM) significantly increased the spare capacity respiration (Figure 6E) and proton leak (Figure 6F) in hGF-1 cells compared with the control group. Treatment with 0.1, 1, and 10 μM (+)-rhodoptilometrin significantly increased ECAR in hGF-1 cells compared with the control group cells (Figure 6G). Therefore, these results showed that (+)-rhodoptilometrin can significantly improve mitochondrial OXPHOS (basal respiration, ATP production, maximal respiration, spare capacity respiration, and proton leak) and glycolytic (ECAR) function.

### 2.7. Effects of (+)-Rhodoptilometrin on the Protein Expression Level of Mitochondrial Complexes I–V in hGF-1 Cells

**Figure 7:**
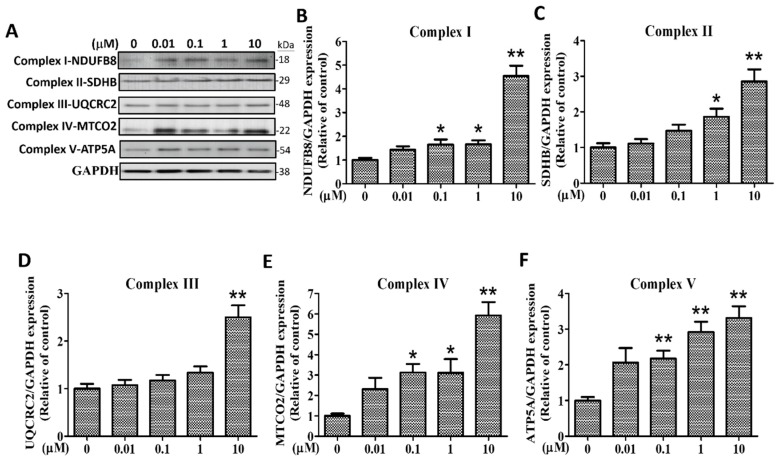
Effects of (+)-rhodoptilometrin on the expression of mitochondrial complexes I~V in hGF-1. (**A**) Complex I, II, III, IV, and V expression in hGF-1 cells was evaluated by western blot analysis after treatment with various dosages of (+)-rhodoptilometrin for 24 h. GAPDH was detected as the internal control. Complex I-NDUFB8 (**B**), complex II-SDHB (**C**), complex III-UQCRC2 (**D**), complex IV-MTCO2 (**E**), and complex V-ATP5A (**F**) levels were quantified by densitometry analysis using ImageJ software and were normalized with the level of GAPDH, expressed as a normalization of the control. Data (means ± SEM) are representative of at least three independent experiments. Student’s *t*-test determined the significance; * *p* < 0.05, ** *p* < 0.01, compared with untreated cells.

OXPHOS is a metabolic pathway in cells. This process occurs in the eukaryotic electron transport chain to form a proton gradient on the mitochondrial inner membrane, which drives the synthesis and release of adenosine triphosphate (ATP) energy through chemical components. To study the effects of (+)-rhodoptilometrin and its relationship with OXPHOS, we employed western blotting analysis to determine the protein expression levels of subunits of respiratory enzyme complexes (complex I, NDUFB8-NADH dehydrogenase (ubiquinone) 1 beta subcomplex, 8, 18kDa; complex II, SDHB-Succinate dehydrogenase complex, subunit B, iron sulfur (Ip), 29 kDa; complex III, UQCRC2-Ubiquinol-cytochrome c reductase core protein II, 48 kDa; complex IV, MTCO2- cytochrome c oxidase subunit 2 family, 22 kDa; and complex V, ATP5A-Mitochondrial membrane ATP synthase, 54 kDa). Figure 7A shows the five complex protein band profiles with GAPDH (38 kDa) as an internal control protein. After using the quantitative analysis of bands, we found that, compared with the control group, treatment with 0.1, 1, and 10 μM (+)-rhodoptilometrin significantly increased the protein expression levels of complex I-DNUFB8 (Figure 7B), complex IV-MTCO2 (Figure 7E), and complex V-ATP5A (Figure 7F); treatment with 1 and 10 μM (+)-rhodoptilometrin significantly increased the protein expression level of complex II-SDHB (Figure 7C); treatment with 10 μM (+)-rhodoptilometrin significantly increased the protein expression level of complex III-UQCRC2 (Figure 7D). The above results showed that (+)-rhodoptilometrin can increase the protein expression levels of mitochondrial respiratory chain complexes I~V in hGF-1 cells, thereby enhancing OXPHOS and ATP yield.

## 3. Discussion

GR is a pathological state in which gum tissue loss or retraction of the gingival margin results in exposure of the roots of the teeth. Many studies showed that promoting gingival cell proliferation may ameliorate GR [38,39,40,41]. In this study, we found that (+)-rhodoptilometrin can significantly improve proliferation in human gingival cells. However, (+)-rhodoptilometrin has no significant effects on oral mucosa cells. In addition, we found that (+)-rhodoptilometrin can significantly increase the messenger RNA (mRNA) expression level of type I collagen in human gingival cells. Therefore, we can see from the results of this study that the marine compound (+)-rhodoptilometrin has similar functions to an enamel matrix derivative that was used in previous studies on gingival proliferation [42]. Moreover, (+)-rhodoptilometrin acts specifically on gingival fibroblasts and does not affect the proliferation of oral mucosa cells. Therefore, (+)-rhodoptilometrin has the developmental potential for clinical applications.

The ocean has rich biodiversity and contains many resources, providing countless possibilities for future drug development. The marine natural compound rhodoptilometrin is an anthraquinone that was extracted and purified from *H. magnipinna*. In a previous study in 2009, Wright et al. employed NMR to demonstrate that rhodoptilometrin exists as two stereoisomers (*S*- or *R*-configuration) [34]. In our previous study, we applied X-ray crystallography to confirm that the rhodoptilometrin isolated from *H. magnipinna* is presented as the *R*-configuration, i.e., (+)-rhodoptilometrin [35]. It also showed significant inhibitory effects on pro-inflammatory protein, inducible nitric oxide synthase, in lipopolysaccharide-stimulated murine macrophages. Moreover, a previous study showed that (+)-rhodoptilometrin exhibits significant cytotoxicity toward the MCF-7 breast cancer cell line, SF-268 glioblastoma cell line, and the H460 non-small-cell lung cancer cell line [34].

Previous studies found that rinsing with physiological saline can promote wound healing and migration in hGF-1 human gingival fibroblast. That study employed an MTT assay to analyze the effects of physiological saline on proliferation in human gingival cells, and the results showed that physiological saline has no effects on the proliferation of human gingival cells [14]. Huynh et al. report that physiological saline can significantly increase the gene expression levels of migration-associated markers (type I collagen, fibronectin, FAK) in human gingival cells; also, immunofluorescence staining showed that physiological saline causes FAK to be located around fibroblasts, whereas F-actin units are arranged in dense parallel arrays of bundles [14]. These results of that study showed that increasing the expression levels of ECM proteins in human gingival cells can promote cell migration and enhance wound healing [14]. In the present study, we employed the MTT assay, scratch-test assay, and transwell migration assay to evaluate the effects of different (+)-rhodoptilometrin concentrations on human gingival cells and oral mucosa cells; however, our results showed that (+)-rhodoptilometrin can promote cell viability, increase the number of migrating cells, and improve wound-healing rates of human gingival cells, but has no effect on oral mucosa cells. In addition, real-time PCR and western blotting was employed to assess the gene and protein expression levels of migration-associated molecular markers, respectively. We found that (+)-rhodoptilometrin increases the gene and protein expression levels of FAK, fibronectin, and type I collagen in human gingival cells. On the other hand, cell immunofluorescence staining also showed that (+)-rhodoptilometrin increases the protein expression levels of F-actin and FAK in human gingival cells. Moreover, treatment with the FAK inhibitor PF-562271 could significantly inhibit the wound-healing effects of (+)-rhodoptilometrin on human gingival cells. The results of this study showed that (+)-rhodoptilometrin not only promotes proliferation, migration, and wound healing in human gingival cells, but also significantly increases the expression levels of migration-associated markers. Our research drug (+)-rhodoptilometrin was similar to rinsing with physiological saline; the only difference is that, in our study, (+)-rhodoptilometrin affected cell viability, while physiological saline does not affect cell proliferation [14]. Therefore, (+)-rhodoptilometrin is expected to be applied in relevant studies on cell migration and wound healing in the future and possesses potential for treating GR.

FAK has a central role in modulating the maturation and stability of focal adhesions and is regarded as the central hub for integrin signaling transduction [43]. A previous study showed that FAK-knockout mice exhibit early embryonic death, and these embryos show severe mesodermal deficiency. In addition, FAK-knockout fibroblasts from these mice also exhibit severe cell migration defects. This indicated that FAK plays an important role in promoting cell migration [44]. Another study showed that microinjection of an FAK C-terminal recombinant protein, such as FAK-related non-kinase (FRNK), inhibits FAK activation and reduces fibroblast migration [45]. In contrast, overexpression of FAK promotes the cell migration of fibronectin [46,47]. FAK can also regulate cell assembly and the actin cytoskeleton to regulate cell migration. However, FAK deletion decreases cell migration, but this can be rescued by FAK overexpression [48,49]. In this study, we found that (+)-rhodoptilometrin promoted migration in hGF-1 cells and also significantly increased the gene and protein expression levels of FAK. Based on these results, it is well demonstrated that FAK plays an important role in (+)-rhodoptilometrin-induced cell migration, potentially providing a promising perspective in the future for its application to studies on cell migration.

Recently, the importance of mitochondria in cell migration became a research hotspot [50,51,52]. A study showed that increased mitochondrial activity and increased distribution to the cytoskeleton are intimately associated with cell migration [53]. The mitochondrion is the main organelle in the cell that generates ATP, which provides energy for metabolism and biosynthesis. There are two routes via which cells produce ATP, namely OXPHOS and glycolysis. The electron transport chain on the inner mitochondrial membrane contains complexes I, II, III, IV, and V, which are essential to the maintenance of proton gradients for energy production in the mitochondria [54,55]. Recently, a study showed that ATP production through glycolysis and mitochondrial OXPHOS could support cell mobility functions such as adhesion, migration, proliferation, and differentiation [56]. The results of our study showed that (+)-rhodoptilometrin increases glycolysis and OXPHOS activity in human gingival cells, including basal respiration, ATP production, maximum respiration, proton leak, spare capacity respiration, and ECAR. Therefore, (+)-rhodoptilometrin increases the activity of complexes I~V in the electron transport chain and also significantly increases mitochondrial respiration. This is the first paper suggesting that (+)-rhodoptilometrin can promote mitochondrial OXPHOS and glycolysis in human gingival cells.

Crinoid-derived (+)-rhodoptilometrin can promote proliferation and migration of human gingival fibroblasts, but does not affect oral mucosa fibroblasts. Hence, (+)-rhodoptilometrin has good potential for future clinical applications. These applications include periodontal surgery and dental implant surgery, which are often accompanied by postoperative complications such as GR and incomplete healing. Furthermore, aging generally leads to gingival recession, resulting in decreased teeth protection and poor appearance. In the future, (+)-rhodoptilometrin may be used as a surgical additive in dental surgery, ointments, and mouthwashes to improve wound healing and the viability and proliferation of gingival cells. Lastly, it can also be applied to non-surgical cosmetic medicine. Therefore, it has the developmental potential for dental healthcare applications.

## 4. Materials and Methods

### 4.1. Chemical

The marine compound (+)-rhodoptilometrin is an anthraquinone that was isolated from a crinoid, *Himerometra magnipinna*, and was provided by P.-J.S. and J.-H.S. from the National Museum of Marine Biology and Aquarium.

### 4.2. Cell Culture

The hGF-1 human gingival fibroblast cell line was obtained from the American Type Culture Collection (ATCC^®^ CRL-2014™ *Homo sapiens* gingival biopsy normal, Manassas, VA, USA), and the oral mucosa fibroblast (OMF) cells were obtained from Professor Michael Hsiao from Academia Sinica. The cells were grown in Dulbecco’s modified Eagle’s medium (DMEM) (Invitrogen Corporation, Carlsbad, CA, USA) supplemented with 10% heat-inactivated fetal bovine serum (FBS), 2 mM glutamine, 1 mM pyruvate, 4.5 g/L glucose, 50 U/mL penicillin, and 50 μg/mL streptomycin in an incubator with conditions of 37 °C, 5% CO_2_, and 95% air.

### 4.3. Cell Viability Experiment (MTT Assay)

In the MTT assay, succinate dehydrogenase (SDH) and cytochrome c in the mitochondria convert yellow 3-(4,5-dimethylthiazol-2-yl)-2,5-diphenyl tetrazolium bromide (MTT, #298-931, USB Corporation, Cleveland, OH, USA) to purple formazan, which is used to analyze cell viability. Cells were seeded on a 24-well plate at 2 × 10^4^ cells per well and cultured for 24 h; then, the culture medium was changed to a serum-free culture medium for 12 h. After (+)-rhodoptilometrin (0.01, 0.1, 1, or 10 μM) was added and cells were further cultured for 24 h, 20 μL of 0.5% MTT solution was added, and the plates were incubated for 3 h at 37 °C. The supernatant was discarded, and dimethyl sulfoxide (DMSO) was added to dissolve formazan. Finally, an ELISA reader (epoch, Bio Tek Instruments, Inc., Winooski, VT, USA) was used to measure the absorbance at a wavelength of 570 nm. Differences in the inter-group absorbance values represent cell numbers. The formula used was as follows:

((absorbance value of experimental group − absorbance value of background value)/(absorbance value of control group − absorbance value of background value)) × 100%

### 4.4. Wound-Healing Measurement (Scratch-Test Assay)

After cells were seeded in 24-well plates (50,000 cells per well) and cultured for 24 h, the culture medium was changed to a serum-free culture medium for 12 h, and a 10-μL tip was used to make a scratch across the cell monolayer at the bottom of each well. Subsequently, phosphate-buffered saline (PBS) was used to wash the cells; then 0, 0.01, 0.1, 1, and 10 μM (+)-rhodoptilometrin was added, and the cells were further cultured for another 24 h. At 0, 12, and 24 h, the inverted phase-contrast microscope and digital camera were used to observe the same location and record the “wound region”. The ImageJ analysis software (version 1.50d, National Institutes of Health, Bethesda, MD, USA) was used to analyze the “wound-healing” area, and the residual “wound” regions were compared among groups.

### 4.5. Transwell Migration Assay

Cells were treated with trypsin to form a suspension in serum-free culture medium. Firstly, 200 μL of cells was added to 0, 0.01, 0.1, 1, or 10 μM (+)-rhodoptilometrin and seeded in a 24-well transwell insert (#3422, BD Falcon^TM^ Cell Culture Inserts, BD Biosciences, Bedford, MA, USA) (1 × 10^5^ cells per well). The transwell inserts were transferred to new 24-well plates, which contained serum culture medium, and the transwell migration assay was only carried out after culturing for 12 h. A cotton bud was used to carefully remove non-migrated cells from the upper layer of the membrane, whereas cells that had migrated to the other side of the membrane were fixed with methanol for 10 min. Afterward, cells were stained with 1.4% crystal violet for 30 min before washing with distilled water. In the transwell migration assay, the ImageJ analysis software was used to evaluate the number of migrated cells within the microscope images of five randomly selected regions on every transwell insert. This experiment was used to compare the number of migrated cells and the cell area among groups.

### 4.6. Quantitative Real-Time PCR Analysis

The hGF-1 cells were treated with various concentrations of (+)-rhodoptilometrin for 24 h, and washed with ice-cold PBS; then, total RNA was extracted using the Qiagen RNeasy Mini kit according to the manufacturer’s instructions. The RNA performed reverse transcription using an iScript complementary DNA (cDNA) Synthesis Kit (Bio-Rad, Hercules, CA, USA) to synthesize cDNA according to the manufacturer’s protocol. After mixing the cDNA and specific forward/reverse primers with iQ SYBR Green Supermix (Bio-Rad) and nuclease-free water, the quantitative real-time PCR (qRT-PCR) analysis was carried out using a CFX-96 real-time PCR system (Bio-Rad). The relative amounts of mRNA were calculated using the standard curve method, and were normalized to the housekeeping gene, *GAPDH*. The primers for all genes tested (see Table 1) were designed using Primer 3 software, from Integrated DNA Technologies. The relative gene expression level of the target gene was calculated using the following formula:

mRNA relative expression = 2^−ΔΔCt^ = 2^−(ΔCt(gene) − ΔCt(control))^,

where Ct is the threshold cycle, and ΔCt is the difference between Ct_(gene)_ and Ct_(GAPDH)_.

### 4.7. Cell Protein Analysis

Firstly, hGF-1 cells were treated with (+)-rhodoptilometrin for 24h. The cells were then washed with ice-cold phosphate-buffered saline (PBS), and lysed in ice-cold lysis buffer (50 mM Tris, pH 7.5, 150 mM NaCl, 1% Triton X-100, 100 μg/mL phenylmethylsulfonyl fluoride, 1 μg/mL aprotinin). The resulting homogenate was centrifuged at 20,000× *g* for 30 min at 4 °C. The supernatant was taken carefully from the pellet, and analyzed as the total protein extract. The supernatant was reserved for western blot analysis. Protein concentrations were determined using the DC protein assay kit (Bio-Rad, Hercules, CA, USA) modified from the method of Lowry et al. [57]. The supernatants containing protein extraction from hGF-1 cells were mixed with equal volumes of sample buffer (2% sodium dodecyl sulfate (SDS), 10% glycerol, 0.1% bromophenol blue, 2% 2-mercaptoethanol, and 50 mM Tris–HCl, pH 7.2). The mixture was then loaded onto an SDS polyacrylamide gel and electrophoresed at 150 V for 90 min. The proteins were transferred to a polyvinylidene difluoride membrane (PVDF; Immobilon-P, Millipore, 0.45-μM pore size) at 125 mA overnight at 4 °C in transfer buffer (50 mM Tris–HCl, 380 mM glycine, 1% SDS, and 20% methanol). After blocking for 40 min with 5% non-fat dry milk in Tris-buffered saline with Tween (TBST) (0.1% Tween 20, 20 mM Tris–HCl, 137 mM NaCl, pH 7.4), the membrane was incubated for 120 min at room temperature with the following primary antibodies: anti-type I collagen (Ab34710), anti-complex I subunit-NDUFB8 (Ab110242), anti-complex II subunit-SDHB (Ab14714), anti-complex III subunit-UQCRC2 (Ab14745), anti-complex IV subunit-MTCO2 (Ab110258), anti-complex V subunit-ATP5A (Ab14748) (all from Abcam, Inc.), anti-FAK (ZF002), and anti-fibronectin (FBN11) (both from Thermo Fisher Scientific Inc.). After the membrane was washed three times for 10 min each with TBST buffer, it was incubated in the appropriate horseradish peroxidase (HRP)-conjugated secondary antibody at room temperature for 120 min. Images were obtained using the UVP BioChemi Imaging System (UVP LLC, Upland, CA, USA), and the relative densitometry was quantified by using LabWorks 4.0 software (UVP LLC). The membranes were re-probed with monoclonal antibodies against anti-GAPDH (G8795, Sigma-Aldrich, Inc., St. Louis, MO, USA) as the internal control for protein loading.

### 4.8. Immunofluorescence Chemical Staining of Cells

Firstly, 1.2 × 10^5^ hGF-1 cells were seeded on coverslips and 0, 0.01, 0.1, 1, or 10 μM (+)-rhodoptilometrin was added into the medium for 24 hr. The cells were fixed with 4% formalin–PBS for 10 min. The cells were then washed three times with PBS buffer and incubated with blocking solution containing 4% goat serum containing 0.01% Triton X-100. After blocking, specific primary antibodies (anti-FAK) were incubated for 2 h at room temperature before PBS washes for removal of excessive solutions. Specific secondary antibodies (Alexa 546-conjugated secondary antibodies (red) and F-actin Alexa Fluor 488 Phalloidin (green) were then applied for further labeling. For immunostaining analysis, the stained sections were examined under a Leica TCS SP II confocal microscope (Leica Instruments Inc., Wetzlar, Germany).

### 4.9. Measurement of Mitochondrial Function

The Seahorse XF24 Extracellular Flux Analyzer (Seahorse Bioscience Inc., Chicopee, MA, USA) was used to measure OCR and ECAR of mitochondria and cells. For inter-experiment comparison, the data were expressed as pMoles/min/2 × 10^4^ cells for OCR and mpH/min/2 × 10^4^ cells for ECAR. The 24-well Seahorse XF cell culture microplates (2 × 10^4^ cells per well) were used in triplicate. After incubation in the incubator overnight, 0, 0.01, 0.1, 1, and 10 μM (+)-rhodoptilometrin was used to treat cells for 24 h. Sodium bicarbonate-free DMEM (pH = 7.4) was used to wash the cells before 675 μL DMEM was added to each well in the 24-well plate. Baseline OCR was measured four times under baseline conditions, and the graph of a function was plotted. Following that, the Seahorse XF Cell Mito Stress Test kit (Seahorse Bioscience Inc., Chicopee, MA, USA), which includes 1 μM oligomycin, 0.5 μM carbonyl cyanide-4-(trifluoromethoxy) phenylhydrazone (FCCP), and 1 μM rotenone/antimycin A, was used. After recording ended, cells were collected, and trypan blue was used to count the cells. After the cell number was standardized, OCR and ECAR values were calculated.

### 4.10. Statistical Analysis

All experimental data were expressed as means ± standard error of the mean (SEM). The inter-group comparison was carried out using the *t*-test. A *p*-value of <0.05 was considered to be significant, and statistical analysis was carried out.

## 5. Conclusions

The marine-derived anthraquinone, rhodoptilometrin, increases FAK expression and promotes mitochondrial function to affect cell migration and promote gingival regeneration. It may be a promising therapeutic agent for GR.

## Figures and Tables

**Table 1:** Human gene primers used in qRT-PCR.

Name	Gene No.	Gene Length (bps)	Primer Sequence 5′–3′	Amplicon (bps)	Annealing Temperature
COL1	NM_000088.3	4393	F: GTGAACCCGGACCCACTG	203	60
			R: CAGACCCTTGGCACCAGG		
FAK	NM_005607.4	3225	F: GAAGCCTTGCCAGCCTCA	183	60
			R: GTGGGGCTGGCTGGATTT		
Fn	NM_002026.2	7068	F: GTCAGCCCAACTCCCACC	209	60
			R: TTGGTGGCCGTACTGCTG		
GAPDH	NM_002046	1401	F: CAATGCCTCCTGCACCACCA	175	60
			R: GATGTTCTGGAGAGCCCCGC		

COL1: type I collagen; FAK: focal adhesion kinase; Fn: fibronectin; GAPDH: Glyceraldehyde 3-phosphate dehydrogenase.
